# Longitudinal stability of data-driven fibromyalgia phenotypes across repeated clinical assessments

**DOI:** 10.1097/PR9.0000000000001472

**Published:** 2026-08-05

**Authors:** Kevin V. Hackshaw, Michelle M. Osuna-Diaz, Katherine R. Sebastian, Lianbo Yu, Zhanna Mikulik, M. Monica Giusti, Luis Rodriguez-Saona

**Affiliations:** a Division of Rheumatology, Department of Internal Medicine, Dell Medical School, The University of Texas at Austin, Austin, TX, USA; b Department of Internal Medicine, Dell Medical School, The University of Texas at Austin, Austin, TX, USA; c Center of Biostatistics and Bioinformatics, The Ohio State University, Columbus, OH, USA; d Division of Immunology and Rheumatology, Department of Internal Medicine, The Ohio State University, Columbus, OH, USA; e Department of Food Science and Technology, The Ohio State University, Columbus, OH, USA

**Keywords:** Fibromyalgia, Chronic pain, Symptom phenotyping, Longitudinal stability, Central sensitization, Functional impairment

## Abstract

**Introduction:**

Fibromyalgia is a heterogeneous chronic condition characterized by variability in pain, psychological distress, central sensitization, and functional impairment. Cross-sectional studies have identified reproducible symptom phenotypes; however, their longitudinal stability remains incompletely understood. **Objectives:**This study aimed to evaluate the longitudinal stability of data-driven fibromyalgia phenotypes across repeated clinical assessments in a large, harmonized, multi-institutional cohort.

**Methods:**

We analyzed longitudinal data from 821 adults with fibromyalgia recruited from 2 academic medical centers. Standardized assessments included the Revised Fibromyalgia Impact Questionnaire, Beck Depression Inventory, Central Sensitization Inventory, McGill Pain Questionnaire, and selected SF-36 functional domains. Unsupervised clustering was performed at baseline to derive symptom phenotypes, which were ordered by overall severity. Using baseline-derived model parameters, phenotype membership was assigned at subsequent visits. Phenotype transitions were examined between visit 1 and visit 2 (n = 191) and across visits 1 to 3 (n = 72) using transition matrices and alluvial visualizations.

**Results:**

Two clinically interpretable fibromyalgia phenotypes were identified at baseline, representing lower-severity/preserved function and higher-severity/global impairment profiles. Across 6-month follow-up intervals, most participants remained within their baseline phenotype (61.9% from visit 1→2 and 64.8% from visit 2→3). Transitions occurred in a minority of individuals and were primarily between adjacent severity phenotypes. Among participants with 3 or more visits, phenotype membership remained stable across repeated assessments.

**Conclusion:**

Data-driven fibromyalgia phenotypes demonstrate substantial longitudinal stability, supporting their validity as enduring clinical constructs rather than transient symptom states. These findings support phenotype-based stratification for longitudinal research and precision treatment approaches in fibromyalgia. Longitudinal analysis shows that fibromyalgia symptom phenotypes are largely stable over time, with infrequent transitions occurring mainly between adjacent severity groups.

## Abstract 2

Longitudinal analysis shows that fibromyalgia symptom phenotypes are largely stable over time, with infrequent transitions occurring mainly between adjacent severity groups.

## 1. Introduction

Fibromyalgia (FM) is a chronic pain disorder characterized by widespread pain, heightened sensory sensitivity, psychological distress, fatigue, and impaired physical and social functioning.^2,5,15,19,37^ Despite its prevalence and clinical impact, fibromyalgia remains diagnostically and therapeutically challenging, in part due to marked interindividual heterogeneity in symptom expression and disease burden.^13,16,20,23,24,29^ This heterogeneity has complicated efforts to identify mechanistic subgroups, predict outcomes, and personalize treatment strategies.^7,11,14^

In recent years, data-driven approaches have been increasingly applied to fibromyalgia to move beyond binary diagnostic classifications toward empirically derived symptom phenotypes. Unsupervised clustering and dimensionality-reduction techniques applied to multidomain clinical data have consistently identified reproducible fibromyalgia subgroups distinguished by overall symptom burden, affective distress, central sensitization, and functional impairment.^6,12,21,28,31^ These phenotypes appear robust across cohorts and analytic methods and may reflect underlying differences in neurobiological vulnerability, psychosocial context, or illness trajectory.

However, most prior phenotyping studies have been cross-sectional, limiting inference about whether identified phenotypes represent stable clinical states or fluctuate substantially over time.^8,10,38^ Longitudinal stability is a critical criterion for the validity and utility of any phenotypic classification system. If symptom phenotypes shift unpredictably across short time intervals, their value for prognostication, mechanistic studies, and treatment stratification would be limited. Conversely, stability would support their interpretation as enduring illness profiles with potential biological relevance.

The present study addresses this gap by evaluating the longitudinal stability of data-driven fibromyalgia phenotypes in a large, harmonized, multisite cohort with repeated assessments over time. Leveraging standardized clinical measures and prospective follow-up, we examined phenotype persistence and transition across sequential visits. We hypothesized that fibromyalgia phenotypes derived at baseline would demonstrate substantial stability over time, with transitions occurring in a minority of individuals and primarily between adjacent severity groups.

## 2. Methods

### 2.1. Ethical approval

All studies involving human participants were approved by the Ohio State University Institutional Review Board (IRB #2015H0312) and conducted in accordance with the principles of the Declaration of Helsinki. The University of Texas at Austin Institutional Review Board also approved the study (IRB #2020030008). All participants provided written informed consent before participation.

### 2.2. Study population and data sources

Participants were drawn from 2 U.S. academic medical centers and included adults meeting diagnostic criteria for fibromyalgia.^1,35,36^ Data from both institutions were harmonized to create a unified longitudinal dataset (Table 1). Participants completed standardized clinical assessments at baseline and at follow-up visits conducted approximately 6 months apart. The present analysis focused on individuals with fibromyalgia and excluded participants with overlapping inflammatory or autoimmune diagnoses for primary analyses.

**Table 1:** Combined cohort composition and longitudinal follow-up.

Dx	Unique participants	Total observations	Participants Ge 2	Participants Ge 3	Median follow-up months
FM	836	1129	194	75	6
HC	75	75	0	0	
LBP	42	43	1	0	6
OA	74	82	6	1	6
RA	193	240	40	9	6
SLE	128	149	16	4	6

Fibromyalgia (FM), Healthy Control (HC), Low Back Pain (LBP), Osteoarthritis (OA), Rheumatoid Arthritis (RA), Systemic Lupus Erythematosus (SLE).

### 2.3. Clinical measures

Clinical assessments included the Revised Fibromyalgia Impact Questionnaire (FIQR) as a global measure of fibromyalgia severity,^5^ the Beck Depression Inventory (BDI) for depressive symptoms,^3,4^ the Central Sensitization Inventory (CSI),^26^ the McGill Pain Questionnaire (MPQ),^9,22^ and selected Short Form-36 (SF-36) domains assessing physical functioning, bodily pain, vitality, and social functioning.^32^ Higher scores on FIQR, BDI, CSI, and MPQ reflect greater symptom burden, whereas higher SF-36 scores indicate better health status. Descriptive statistics for these measures at baseline across phenotypes are summarized in Table 2.

**Table 2:** Baseline characteristics (visit 1 only)—fibromyalgia cohort.

Baseline fibromyalgia phenotypes defined by K-means clustering					
Phenotype	N	FIQR mean (SD)	BDI mean (SD)	CSI mean (SD)	MPQ mean (SD)
Lower-severity phenotype	455	40.2 (15.2)	15.5 (8.2)	54.7 (11.9)	64.6 (29.8)
Higher-severity phenotype	366	68.0 (13.2)	30.5 (10.8)	75.5 (12.3)	136.4 (35.2)
Total N = 821					

BDI, Beck Depression Inventory; CSI, Central Sensitization Inventory; FIQR, Fibromyalgia Impact Questionnaire; MPQ, McGill Pain Questionnaire.

### 2.4. Phenotype derivation

**Figure 1.**
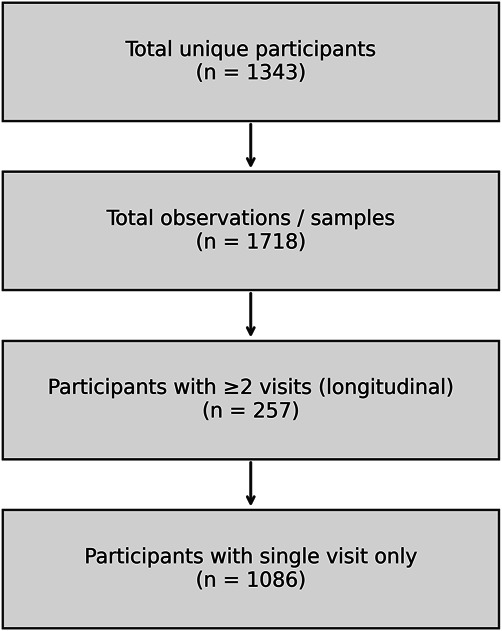
Observational cohort flow and longitudinal follow-up. Flow diagram illustrating participant inclusion, exclusion, and longitudinal follow-up in the fibromyalgia cohort. A total of 821 participants completed baseline assessment (visit 1). Of these, 191 participants completed at least one follow-up visit and 72 participants completed 3 or more visits. Follow-up assessments were conducted approximately 6 months apart. Longitudinal analyses focused on visit 1→visit 2 and visit 1→visit 2→visit 3 transitions.

Unsupervised clustering was performed using baseline (visit 1) data only to derive fibromyalgia phenotypes. Before clustering, SF-36 domains were sign-inverted so that higher values consistently reflected greater symptom burden across all measures. Missing values were imputed using median imputation, and all variables were standardized to z-scores. A 2-cluster solution was selected based on silhouette statistics and clinical interpretability. Phenotypes were ordered by baseline FIQR severity and labeled as lower-severity/preserved function and higher-severity/global impairment (Fig. 1).

### 2.5. Longitudinal phenotype assignment

Baseline-derived imputation parameters, scaling factors, and cluster centroids were applied to follow-up visits to assign phenotype membership at subsequent time points using nearest-centroid classification. This approach ensured that longitudinal changes reflected within-individual symptom evolution rather than reestimation of phenotype structure at each visit as detailed in the methods.

### 2.6. Statistical analysis

**Figure 2.**
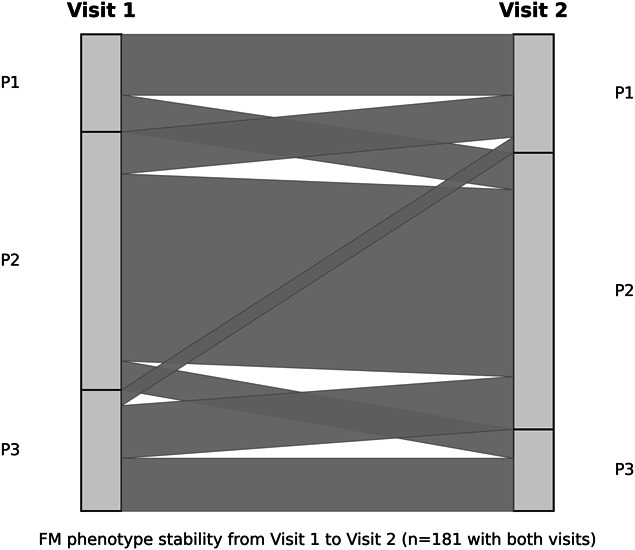
Longitudinal stability of fibromyalgia phenotypes across sequential visits. Alluvial diagrams depicting transitions between data-driven fibromyalgia phenotypes across sequential visits from visit 1 to visit 2 (left panel) and from visit 2 to visit 3 (right panel). Phenotypes were derived at baseline and ordered by overall symptom severity as lower-severity/preserved function and higher-severity/global impairment. Bar heights represent the proportion of participants within each phenotype at each visit, and ribbon widths are proportional to the number of individuals transitioning between phenotypes. Percentages indicate the proportion of participants within each analytic cohort (visit 1→visit 2: n = 191; visit 1→visit 2→visit 3: n = 72).

Phenotype stability was assessed by constructing transition matrices for visit 1→visit 2 and visit 2→visit 3 among participants with available data at both visits. Transition probabilities were row-normalized to estimate the likelihood of remaining in the same phenotype vs transitioning to another phenotype. Longitudinal trajectories across 3 visits were summarized descriptively. Transitions were visualized using alluvial diagrams, with ribbon widths proportional to the number of individuals transitioning between phenotypes (Fig. 2).

## 3. Results

### 3.1. Cohort characteristics and longitudinal follow-up

A total of 836 participants with fibromyalgia were available in the harmonized dataset (Table 1). Of these, 823 participants had complete baseline clinical data and were included in baseline descriptive analyses (Table 2). Two additional participants were excluded from clustering due to incomplete data, yielding 821 individuals for phenotype derivation (Fig. 1). Of these, 191 participants (23.3%) completed at least one follow-up visit, and 72 participants (8.8%) completed 3 or more visits (Fig. 1). Baseline demographic and symptom characteristics were broadly comparable between participants who completed follow-up visits and those who did not, with no clinically meaningful differences observed in baseline FIQR, BDI, CSI, or MPQ scores. A smaller subset contributed extended longitudinal data across additional visits. Longitudinal analyses focused on visit 1→visit 2 and visit 1→visit 2→visit 3 transitions, which provided sufficient sample size for stable estimation.

### 3.2. Baseline fibromyalgia phenotypes

At baseline, 2 distinct fibromyalgia phenotypes were identified (Table 2). Mean symptom scores differed substantially across clusters, with FIQR scores averaging 40.2 (SD 15.2) in the lower-severity phenotype and 68.0 (SD 13.2) in the higher-severity phenotype, accompanied by corresponding increases in BDI, CSI, and MPQ scores (Table 2). Mean symptom scores reflected increasing burden across the 2 phenotypes. The low-severity phenotype demonstrated lower FIQR, CSI, BDI, and MPQ scores with relatively preserved SF-36 physical functioning. In contrast, the high-severity phenotype demonstrated substantially elevated symptom scores across all measures and marked functional impairment across pain, mood, central sensitization, and functional domains. Across clusters, FIQR, CSI, BDI, and MPQ scores increased progressively from the low- to high-severity phenotype, supporting the ordered structure of the derived phenotypes. Demographic characteristics including age, sex distribution, and body mass index (BMI) were broadly comparable across clusters and were not primary drivers of cluster formation.

### 3.3. Phenotype stability across visits

Between visit 1 and visit 2, most participants remained within their baseline phenotype, indicating substantial longitudinal stability over approximately 6 months (Fig. 2). Transition matrices further demonstrated high probabilities of remaining within the same phenotype across visits, with lower probabilities of transitioning between nonadjacent phenotypes. Transitions occurred in a minority of participants and were most commonly observed between adjacent severity phenotypes rather than between the lowest- and highest-severity groups. Similar patterns were observed between visit 2 and visit 3 (Fig. 2).

Among participants with 3 or more visits, longitudinal trajectories showed that most individuals maintained consistent phenotype membership across all 3 assessments (Fig. 2). When transitions occurred, they tended to reflect modest shifts in symptom severity rather than wholesale reclassification across the phenotypic spectrum.

## 4. Discussion

In this large, harmonized, multisite cohort, data-driven fibromyalgia phenotypes demonstrated substantial longitudinal stability across repeated clinical assessments (Fig. 2). These findings extend prior cross-sectional phenotyping studies by showing that empirically derived fibromyalgia subgroups persist over time and are not merely transient reflections of short-term symptom fluctuation.^6,12,28,31^ These findings suggest that the identified phenotypes represent reproducible patterns of symptom organization rather than cohort-specific artifacts. Importantly, these phenotypes were defined using multiple symptom domains encompassing pain severity, affective distress, central sensitization, and functional impairment. Although the resulting clusters are ordered along a multidimensional burden continuum, they capture clinically meaningful patterns of symptom expression across domains rather than variation in a single severity metric. This multidomain structure supports their interpretation as clinically informative phenotypes that may reflect underlying differences in disease impact and treatment needs. The predominance of within-phenotype stability, coupled with transitions primarily between adjacent severity groups, supports the interpretation of these phenotypes as enduring clinical profiles.^34,38^ An important conceptual question is whether the derived clusters represent distinct clinical phenotypes or simply ordered levels of symptom severity. In the present study, the clustering structure largely reflected stratification along a multidimensional burden continuum encompassing pain intensity, central sensitization, affective distress, and functional impairment. However, these groupings remain clinically informative because they capture coherent profiles of symptom expression across multiple domains rather than variation in a single measure. Similar burden-structured phenotypes have been reported in prior fibromyalgia and chronic pain phenotyping studies and have been proposed as useful frameworks for patient stratification, prognosis, and treatment selection. From this perspective, the identified clusters may be best understood as severity-structured phenotypes within a multidimensional clinical spectrum rather than entirely discrete disease subtypes.

The observed stability has important implications for fibromyalgia research and care. Stable phenotypes provide a foundation for longitudinal mechanistic studies, facilitate enrichment strategies for clinical trials, and support the development of phenotype-informed treatment approaches. The finding that transitions tend to occur gradually rather than abruptly suggests that symptom burden in fibromyalgia evolves along a continuum, potentially influenced by treatment, psychosocial factors, or comorbidities.^10,18,27,29^

Strengths of this study include the large sample size, multi-institutional harmonization, use of standardized multidimensional assessments, and prospective longitudinal design (Table 1).^5,9,22,26,32,33^ The analytic approach, which anchored phenotype definition at baseline and applied fixed classification parameters longitudinally, minimizes circularity and enhances interpretability. Anchoring phenotype definition at baseline also avoids the methodological ambiguity that can arise when clusters are reestimated independently at each time point, which may lead to shifting cluster structures that complicate longitudinal interpretation. By applying fixed classification parameters across visits, the present approach enables direct assessment of within-individual phenotype stability while minimizing the risk that apparent transitions reflect changes in the clustering solution itself rather than true changes in symptom burden.

Several limitations warrant consideration. Longitudinal follow-up was available for a subset of participants, reflecting the observational nature of the cohort and the fact that follow-up visits were not mandatory study assessments. As a result, attrition over time may introduce potential selection bias. However, baseline symptom measures were broadly similar between participants who returned for follow-up and those who did not, suggesting that the longitudinal subset remained representative of the overall cohort with respect to symptom burden and clinical characteristics.

Importantly, the stability patterns observed in this study were consistent across both the 2-visit and 3-visit analyses, and transitions between phenotypes were relatively uncommon. These findings suggest that the observed stability is unlikely to be driven solely by selective follow-up of a particular subgroup. Nonetheless, future studies with more complete longitudinal retention will be important to confirm these findings and further evaluate the temporal dynamics of fibromyalgia symptom phenotypes (Table 2). Although follow-up intervals were approximately 6 months, variability in visit timing could influence observed transitions. In addition, the study focused on clinical symptom measures and did not incorporate biological or neuroimaging markers, which will be important for future mechanistic validation.^8,17,25,30,31^

In conclusion, fibromyalgia phenotypes derived from multidimensional clinical data are largely stable over time, supporting their validity as meaningful clinical constructs. These results provide a strong rationale for incorporating phenotype-based stratification into longitudinal studies and precision medicine approaches for fibromyalgia. Such stratification may help identify patient subgroups with distinct trajectories of disease burden and treatment responsiveness.

## Disclosures

The authors have no conflict of interest to declare.
